# Prevalence and Comorbidity of Gender Dysphoria in Taiwan, 2010–2019

**DOI:** 10.1007/s10508-022-02500-7

**Published:** 2023-01-24

**Authors:** Kuo-Yu Chao, Chih-Chiang Chou, Ching-I. Chen, Shu-Ru Lee, Wei Cheng

**Affiliations:** 1 Department of Nursing, Chang Gung University of Science and Technology, Taoyuan, Taiwan; 2 Division of Colon and Rectal Surgery, Chang Gung Memorial Hospital, Linkou, Taiwan; 3 Department of Psychiatry, Centro Hospitalar Conde de São Januário, Sé, Macau SAR China; 4 Department of Psychiatry, Keelung Hospital, Ministry of Health and Welfare, Keelung, Taiwan; 5 Research Services Center for Health Information, Chang Gung University, Taoyuan, Taiwan; 6 Department of Pathology, Keelung Hospital, Ministry of Health and Welfare, Keelung, Taiwan; 7 School of Nursing, National Taipei University of Nursing and Health Sciences, Taipei, Taiwan; 8 Department of Nursing, Ching Kuo Institute of Management and Health, Keelung, Taiwan

**Keywords:** Gender dysphoria, Attention-deficit hyperactivity disorder (ADHD), Autism spectrum disorder (ASD), Schizophrenia, Psychosis, Depression

## Abstract

Gender dysphoria (GD) is a condition in which a person exhibits marked incongruence between their expressed or experienced gender and their sex assigned at birth. The last survey of individuals with GD in Taiwan was conducted approximately 10 years ago. In this study, we investigated the prevalence of GD in Taiwan within the last 10 years as well as comorbidities. A retrospective medical record review was performed for all patients in the database of the Health and Welfare Data Science Center covered by National Health Insurance in Taiwan from January 2010 until December 2019. The study population of persons with GD was defined as individuals who had been diagnosed with transsexualism (transgender or transsexual) or gender identity disorders. Our review found case numbers and prevalence of GD in 2019 were about twice that of patients in 2010 for both assigned males and assigned females at birth. Case numbers for 2010 versus 2019 were 440 versus 867 for assigned males at birth, and 189 versus 386 for assigned females at birth. The 1-year prevalence for 2010 versus 2019 was 3.8/100,000 versus 7.4/100,000 for assigned males at birth, and 1.6/100,000 versus 3.2/100,000 for assigned females at birth. Comorbidities of attention-deficit/hyperactivity disorder (ADHD), autism spectrum disorder (ASD), and psychosis were more likely in children with GD younger than 12 years of age; comorbid depression was more likely in adolescents and adults with GD. Improvements in social and mental health support should be provided to help address these comorbidities of ADHD, ASD, and depression among individuals with GD.

**Supplementary Information:**

The online version contains supplementary material available at 10.1007/s10508-022-02500-7.

## Introduction

The fifth edition of the *Diagnostic and Statistical Manual of Mental Disorders* (*DSM-5*) defines “transgender” as “the broad spectrum of individuals who transiently or persistently identify with a gender different from their natal gender. The fifth edition of the DSM was also the first time the term gender dysphoria (GD) was used as a diagnosis, which is defined as a condition in which a person exhibits marked incongruence between sex assigned at birth and their expressed or experienced gender (American Psychiatric Association, 2013; Saleem & Rizvi, 2017). GD differs from gender variance, which is an umbrella term used to describe gender expression or behavior that falls outside of culturally defined norms associated with a specific gender (Simons et al., 2014). Therefore, not all gender-variant individuals experience gender dysphoria (Simons et al., 2014). The condition of GD can cause clinically significant distress or impairment in social, occupational, or other vital areas of functioning. Individuals with GD experience a strong desire to be treated as the other gender (or some alternative gender different from their sex assigned at birth) or to be rid of their sex characteristics. They may also report a strong conviction of having feelings and reactions typical of the other gender (or some alternative gender). The previous diagnostic term, gender identity disorder (GID), was abandoned in *DSM-5* to avoid pathologizing identity (Kaltiala-Heino et al., 2018).

A systematic review and meta-analysis demonstrated an increase in the prevalence of transsexualism over the last 50 years to 4.6 in 100, 000 among adults, with 6.8/100,000 for assigned males at birth and 2.6/100,000 for assigned females at birth (Arcelus et al., 2015). Although GD is considered an uncommon diagnosis, a literature review found an increase in referrals for adolescents to be seen by gender identity specialists (Zucker, 2017). There has also been a significant change from prior years in the adolescent male to female ratio for incongruence of assigned sex for, which previously favored assigned males at birth to a more recent higher proportion of assigned females at birth (Aitken et al., 2015). Studies using short self-reports of gender identity such as gender variance or transgender suggest that 0.17–1.3% of adolescents and young adults identify as transgender (Connolly et al., 2016; Zucker, 2017). Zhang et al., (2020) suggests many reports on the prevalence of individuals who identify as transgender or gender diverse may be higher than reported. When surveys inquire about transgender identity using broader descriptions of what Zhang et al. refer to as ‘gender diversity’, proportions of young adults increased from 0.3–0.5% to 0.5–4.5% and among children and adolescents the proportion increased from 1.2–2.7% to 2.5–8.4% (Zhang et al., 2020).

Although no official clinical data on individuals with GD in Taiwan are available, several nonclinical studies regarding the epidemiological features of GD have been conducted. For example, using the Diagnostic Interview Schedule, one study identified a prevalence of transsexualism in adults ranging from 0.3 to 2.0/1000, with a higher prevalence in assigned females at birth than assigned males at birth (range, 0.7–4.2/1000 vs. 0–0.4/1000) (Hwu et al., 1989). In 2010, a study using questionnaires reported a similar finding in Taiwanese young adults, with a higher prevalence of GD in assigned females at birth (7.3%) than in assigned males at birth (1.9%) (Lai et al., 2010).

Studies have assessed the prevalence of psychiatric disorders in patients seeking treatment for GD (Dhejne et al., 2016; Heylens et al., 2014). With a dramatic increase in referrals for GD since 2002, over half had psychiatric and/or developmental comorbidities confirmed by the Pediatric Endocrinology Clinic of Riley Hospital for Children (Chen et al., 2016). Schizoid personality was strongly associated with GD in young adults in Taiwan (Lai et al., 2010). Attention-deficit hyperactivity disorders (ADHD) and depressive disorders were also common diagnoses for transgender children and adolescents (Becerra-Culqui et al., 2018). An overrepresentation of patients with symptoms of autism spectrum disorder (ASD) and ADHD was observed in individuals with GD compared with the general population (Heylens et al., 2018), and patients referred for ASD or ADHD were more likely to express gender variance than non-referred patients (Strang et al., 2014).

The number of adolescents contacting specialized gender identity services has risen considerably over the past decade across Europe and North America (Littman, 2018; Wood et al., 2013). The ratio of sex favored adolescents who were assigned males at birth until the mid-2000s, when a shift to one favoring adolescents who were assigned females at birth was observed in several countries (e.g., Canada, Netherlands, Finland) (Aitken et al., 2015; Kaltiala-Heino et al., 2015). England has also seen an increase in the number of children considering gender reassignment surgery, which has encouraged the exploration of the role for social media and the teaching of transgender issues in schools (Marchiano, 2021; Rayner, 2018).

In Taiwan, the Gender Equity Education Act was passed in 2004 (Ministry of Education, 2018). In 2012, the Gender Equity Education Act added Article 13, Rules of Enforcement, which included lesbian/gay/bisexual/transgender (LGBT) education (Ministry of Education, 2012). Since then, the educational system has promoted LGBT education, which included replacing terms such as “men and women” with gender-neutral terminology, and increasing sensitivity to the spectrum of gender diversity (Kung, 2020). These changes have been incorporated into educational courses for adolescents to increase knowledge of sexual health (Chao & Cheng, 2021).

Based on the above literature demonstrating an increase in the prevalence of GD in other countries, our study aimed to determine whether the prevalence of GD has also been altered in the Taiwanese population within the last 10 years, especially given the changes in the educational system of Taiwan. In addition, we also investigated the presence of comorbidities of ADHD, ASD, schizophrenia, and depression. Given the increase in prevalence seen in other countries, we hypothesized that prevalence rates of GD in Taiwan would increase in each year from 2010. An understanding of the prevalence of GD in Taiwan and the possible presence of comorbidities in this group could provide an awareness of the increase in the need to provide adequate social and mental health support for individuals with GD.

## Method

### Subjects

This study was approved by the Ethics Committee of Taipei Hospital, Ministry of Health, and Welfare (TH-IRB-0020-0015). All procedures were performed in accordance with the standards of the Ethics Committee of Taipei Hospital, Ministry of Health, and Welfare (MOHW) and with the 1964 Helsinki Declaration and its later amendments or comparable ethical standards.

This retrospective study investigated the prevalence of GD in Taiwan within the last 10 years. Medical data were obtained from the Health and Welfare Data Science Center (HWDC), which was established by the MOHW in 2011. The HWDC is a database containing all health data for the general population of Taiwan according to the codes for International Classification of Disease (ICD), which includes clinical diagnoses and medical, surgical, and psychiatric treatments. Hospitals and medical centers upload all patient data from the National Health Insurance (NHI) program to the HWDC. The NHI is a compulsory insurance policy available to everyone in Taiwan and used by most of the population.

### Measures

For the purposes of this study, when reviewing the HWDC database, we defined individuals with GD as patients who had been diagnosed, according to the ICD code, with transsexualism or a gender identity disorder. In Taiwan, transsexualism is an umbrella term for persons who are transgender and transsexual. ICD codes (described below) are established by the MOHW and confirmed by the Medical Claims Review Board of Taiwan, which is comprised of experts in different medical disciplines of the NHI Administration. If a physician believes a patient’s symptoms involve multiple diagnoses (such as a comorbidity), additional codes are assigned and validated by the appropriate specialty physician.

### Procedure

The retrospective medical record review of the HWDC database used ICD codes to identify all patients evaluated by a psychiatrist and diagnosed with GD from January 2010 until December 2019. Data collected from 2010 to 2015 were based on the ICD-9 diagnostic categories and were defined as transsexualism: 302.50 (transsexualism with unspecified sexual history), 302.51 (transsexualism with asexual history), 302.52 (transsexualism with homosexual history), 302.53 (transsexualism with heterosexual history), 302.6 (GID in children), and 302.85 (GID in adolescents or adults). Data collected for 2016–2019 were based on the ICD-10 diagnostic categories and were all considered as GIDs: F64 (GID), F64.1 (GID in adolescence and adulthood), F64.2 (GID of childhood) F64.8 (other GIDs), and F64.9 (GIDs, unspecified). Assigned sex at birth in the NHI system is determined by the obstetrician-gynecologist or pediatrician. Patients were excluded if there was the presence of a sexual development disorder. We also identified comorbidities of ADHD, ASD, schizophrenia, and depression using the GD codes listed above combined with ICD codes listed in Supplemental Table 1, which were also contained in the database. Because children under the age of 12 years do not receive a diagnosis of “psychosis” in the NHI, in Taiwan ICD codes for schizophrenia are used for all children.

**Table 1:** Number of birth-assigned males and birth-assigned females with gender dysphoria and comorbidities from 2010 to 2019 by age group

	Age (years)						
	≤ 12		13–17		≥ 18		
Assigned sex at birth	Male	Female	Male	Female	Male	Female	Prevalence in the
Gender dysphoria	*N* = 33	*N* = 24	*N* = 299	*N* = 172	*N* = 2464	*N* = 1064	general population^a^

Comorbidities	*n* (%)	*n* (%)	*n* (%)	*n* (%)	*n* (%)	*n* (%)	%
ADHD	7 (21.2)	2 (8.3)	8 (4.0)	4 (2.6)	17 (0.7)	4 (0.4)	^b^5-7
ASD	4 (12.1)	0 (0)	6 (3.0)	1 (0.6)	16 (0.7)	2 (0.2)	1
Schizophrenia/psychosis^c^	2 (6.1)	0 (0)	2 (1.0)	3 (1.7)	34 (1.4)	17 (1.6)	0.4
Depression	2 (6.1)	0 (0)	52 (26.1)	28 (16.3)	564 (22.9)	168 (15.8)	^d^2.7 (Males)/5.0 (Females)

^a^Source: Ministry of Health and Welfare

^b^Prevalence of ADHD in the general population of Taiwan is considered for individuals ≤ 12 years

^c^ A diagnosis of schizophrenia for children ≤ 12 years of age is labeled here as psychosis

^d^Prevalence of depression in the general population of Taiwan is considered for individuals > 15 years of age [excluding individuals ≤ 14 years of age]

### Statistical Analysis

Quantitative data were analyzed using SPSS version 22.0 for Windows (Armonk, NY: IBM Corp). Descriptive statistics were used for frequency (*n*, %). The prevalence was calculated for patients ≤ 12 years of age, 13–17 years of age, and ≥ 18 years of age by using the total number of people in these age groups diagnosed with GD from 2010 to 2019. We used the total populations of these age groups in Taiwan during 2010–2019, which were provided by the Department of Household Registration, Ministry of the Interior of Taiwan, to calculate the 1-year prevalence of GD. The *z*-test assessed differences in the prevalence of depression between patients with GD ≥ 18 years of age and the general population ≥ 15 years of age. Significance was set at *p* < 0.05 for statistical comparisons.

## Results

### Prevalence and Demographics

**Fig. 1:**
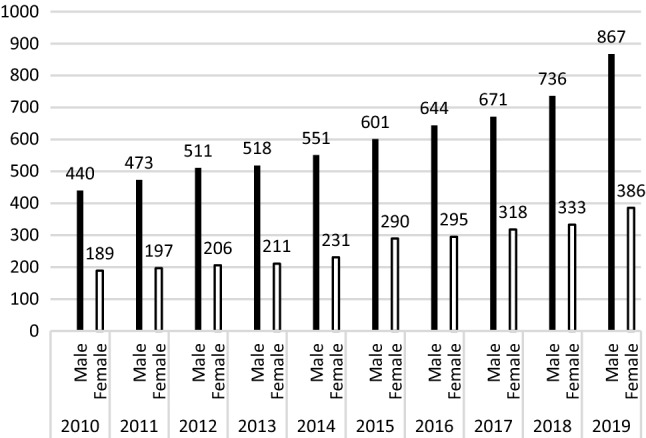
Birth-assigned males and birth-assigned females with gender dysphoria in Taiwan, 2010–2019

The total population of Taiwan is approximately 23,500,000. All patients diagnosed with GD from 2010 to 2019 were included in the analysis. The number of patient cases diagnosed with GD in 2010 compared with 2019 demonstrated a yearly increase from 440 versuss 867 for assigned males at birth, and 189 versus 386 for assigned females at birth (Fig. 1). Notably, the number of patients with GD in 2019 was twice that of patients in 2010 for both patients assigned as males at birth and those assigned as females at birth. Average rates for patients assigned male at birth and total male–female ratios were 2.29:1, higher for patients who were birth-assigned males than birth-assigned females across all timepoints.

**Fig. 2:**
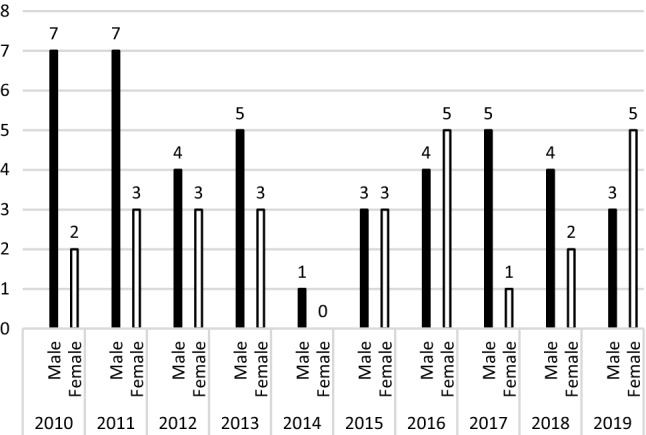
Birth-assigned males and birth-assigned females under 12 years of age with gender dysphoria in Taiwan, 2010–2019

**Fig. 3:**
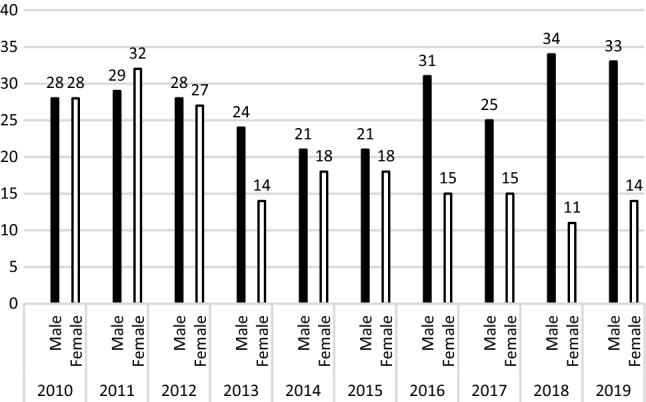
Birth-assigned males and birth-assigned females 13–17 years of age with gender dysphoria in Taiwan, 2010–2019

**Fig. 4:**
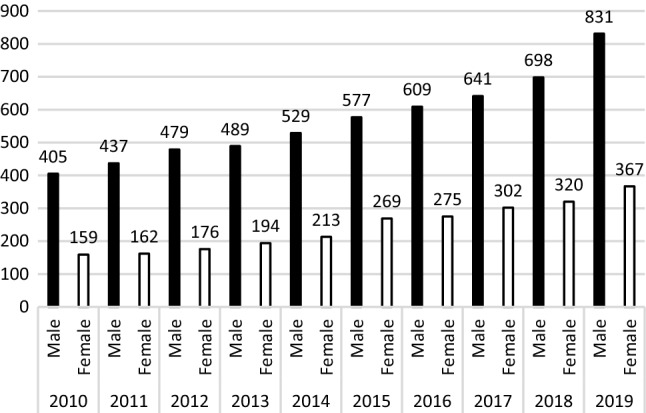
Birth-assigned males and birth-assigned females over 18 years of age with gender dysphoria in Taiwan, 2010–2019

Rates for patients according to age groups are shown in Figs. 2, 3, and 4. For patients ≤ 12 years during the same time, the ratios of birth-assigned boys to birth-assigned girls were 1.38:1; however, in 2016 and 2019 the number of birth-assigned girls with GD was greater than the number of birth-assigned boys with GD (Fig. 2). The male–female ratio was 1.16:1 for 13–17-year-olds, with the number of birth-assigned young males with GD increasing yearly from 2015 (Fig. 3). The male–female ratio for patients ≥ 18 years of age was 2.32:1, and the number of assigned males at birth in 2019 was 2.1 times the number in 2010; the number of assigned females at birth in 2019 was 2.3 times of 2010 (Fig. 4).

The 1-year prevalence of GD for 2010 versus 2019 was 3.8/100,000 versus 7.4/100,000 for assigned males at birth, and 1.6/100,000 versus 3.2/100,000 for assigned females at birth. The 1-year prevalence of GD children under 12 years old was 0.45/100,000 versus 0.21/100,000 for birth-assigned boys, and 0.14/100,000 versus 0.4/100,000 for birth-assigned girls in 2010 and 2019. In GD adolescents of 13–17-year-olds, it was 3.3/100,000 versus 5.7/100,000 for assigned males at birth, and 3.6/100,000 versus 2.7/100,000 for assigned females at birth in 2010 and 2019. In individuals with GD ≥ 18 years of age, the prevalence was 4.3/100,000 versus 8.5/100,000 for assigned males at birth, and 1.7/100,000 versus 3.6/100,000 for assigned females at birth for 2010 versus 2019.

### Comorbidities

We surveyed the prevalence of ADHD, ASD, schizophrenia (psychosis), and depression in patients with GD from 2010 to 2019 (Table 1). The percent of children ≤ 12 years of age with a comorbidity of ADHD was 21.2% in birth-assigned boys and 8.3% in birth-assigned girls. The comorbidities of ASD, schizophrenia (psychosis) and depression present in birth-assigned boys were 12.1%, 6.1% and 6.1%, respectively. None of these three comorbidities were detected in birth-assigned girls. In 13–17-year-olds, the percent of assigned males at birth and assigned females at birth with comorbidities were as follows: ADHD, 4.0% and 2.6%, respectively; ASD, 3.0% and 0.6%, respectively; schizophrenia, 1.0% and 1.7%, respectively; and depression, 26.1% and 16.3%, respectively. In patients with GD ≥ 18 years of age, the percent of assigned males at birth and assigned females at birth with comorbidities were as follows: ADHD, 0.7% and 0.4%, respectively; ASD, 0.7% and 0.2%, respectively; schizophrenia, 1.4% and 1.6%, respectively; and depression, 22.9% and 15.8%, respectively.

We only compared the prevalence of comorbidities between GD who were > 18 and the general population for individuals > 15 years of age, because of the meaningless artifacts of data sparsity for the comorbidities in other GD (Greenland et al., 2016). The sample sizes for other comorbidities were either not enough, or case numbers were minimal and not sufficient for analyses of correlations. The *z*-test indicated a significantly greater prevalence in depression, compared with the general population, for assigned males at birth (*z* = 61.83, *p* < 0.001) and assigned females at birth (*z* = 16.14, *p* < 0.001). Although only comorbidities of depression for patients ≥ 18 years of age are relevant when comparing prevalence to the general population, the depression prevalence was greater for birth-assigned males than birth-assigned females in all three age groups.

## Discussion

In this study, we observed that the number of birth-assigned male and birth-assigned female patients diagnosed with GD increased yearly from 2010 to 2019. Case numbers and prevalence of GD in 2019 were about twice that of 2010 for both assigned males at birth and assigned females at birth. In children ≤ 12 years of age, the number of birth-assigned girls with GD was higher than that of birth-assigned boys in 2016 and 2019; however, it is difficult to draw any conclusions about the prevalence of children with GD ≤ 12 years of age due to the small sample size. Whether this shift indicates a trend in an increase seen in assigned females at birth with GD as has been reported elsewhere (Aitken et al., 2015; Arcelus et al., 2015; Zhang et al., 2020) will require an examination of data over the next 5–10 years. However, in the 13–17-year-old group of children with GD, the general trend appeared to increase slightly for assigned males at birth with GD after 2015. In the group with GD ≥ 18 years of age, the number of assigned males at birth and assigned females at birth had a gradual and sustained increase from 2010 to 2019; by 2019 the total number had risen 2.3-fold for assigned females at birth, and 2.1-fold for assigned males at birth compared with 2010. The overall trend in increase for individuals with GD in Taiwan was similar to increases reported in Western countries.

Our findings indicate a 1.4-fold increase in the number of assigned females at birth from 2010 to 2015 and a 1.5-fold increase in assigned males at birth. The estimated prevalence of GD in Taiwan in 2015 was 2.5/100,000 for assigned females at birth and 5.1/100,000 for assigned males at birth, which reflects a similar increase in transgender individuals in the Netherlands reported by Wiepjes et al. (2018). Individuals diagnosed with GD per year increased about 3.6-fold for assigned females at birth and two fold for assigned males at birth from 2010 to 2015 in the Netherlands with an estimated prevalence of GD in the of 1:5200 (19.2/100,000) for assigned females at birth and 1:3800 (31.3/100,000) for assigned males at birth. Although both the fold increase and prevalence of GD in Taiwan are less than those in the Netherlands, the trend is similar in both countries.

When the case numbers from 2010 to 2019 for age group (Figs. 2, 3, 4) are compared with the overall population (Fig. 1), only the group of birth-assigned males and birth-assigned females ≥ 18 years of age show a similar gradual increase over time. This suggests that for young children (≤ 12 years of age) and adolescents (ages 13–17), GD may be more labile. Our finding for adolescents may be what has been described as “adolescent-onset” GD, which occurs in the early stages of puberty, but is not considered clinically significant (Littman, 2018). Although the origins of GD are multifactorial and may involve genetic or biological mechanisms (Meyer-Bahlburg, 2013; Zucker et al., 2013), twin studies have shown a high concordance of GD in monozygotic twins (Heylens et al., 2012), but no candidate genes have been identified (Boucher & Chinnah, 2020; Claahsen-van der Grinten et al., 2021). However, larger sample sizes for young children and adolescents, and longitudinal studies will be required to examine the relationship more rigorously between an early diagnosis of GD and the percentage of patients for whom this diagnosis is sustained into adulthood.

One reason for the increase in GD in those ≥ 18 years of age may be the recent acknowledgement of the existence of LGBT individuals in Taiwan, which is reflected in more movies oriented toward LGBT young adults and issues of multi-sex/gender issues in Taiwan since 2002 (Lin, 2009, 2010). The Enforcement Rules for the Gender Equity Education Act, announced in 2012, included LGBT education and gender diversity (Ministry of Education, 2012), and then gender spectrum was put in some textbooks (Zhu, 2017). These introduced students to transgender issues as well as the perspective that “multi-sexes/genders are present in our society, gender identity is more important than biological sex,” and “you can choose whatever sex/gender you want to be” (Kung, 2020). Thus, social media, social changes, and changes in the educational system of Taiwan may have helped validate transgender ideation for individuals.

In this study, ADHD was the only disorder that was comorbid in both birth-assigned male and birth-assigned female children with GD ≤ 12 years of age. Comorbidities of ASD, psychosis, and depression were only present in assigned males at birth. One explanation that ADHD was the only comorbidity for assigned females at birth with GD may be due to under-reporting or a small sample size. Therefore, further analysis may be needed accurately assess comorbidities for children with GD in Taiwan. A systematic review of 15 studies children with GD between the ages of 2 and 12 years found the most frequent comorbidities included mood and anxiety disorders, ASD, and self-harm (Frew et al., 2021). The authors suggest international guidelines for assessments and treatment be developed to provide greater consistency in diagnosis and management (Frew et al., 2021).

In adolescents with GD, the greatest frequency was the comorbidity for depression, which was 26.1% for assigned males at birth and 16.3% for assigned females at birth. Although the prevalence of depression in the general population of Taiwan is 2.7% for males and 5.0% for females, we were unable to determine whether these high percentages were significant because of the small sample sizes for patients with GD and depression. However, the high percentage could be explained by victimization (e.g., social stigma, discrimination), difficulties accessing health care and social services, and gender and interpersonal problems that have been demonstrated to put individuals with GD at risk of developing psychiatric disorders, particularly depression (Dhejne et al., 2016).

The second highest frequency for adolescents with GD was ADHD: 4.0% for assigned males at birth and 2.6% for assigned females at birth. A Strang et al. (2014) observed that children with ADHD were 6.64 times more likely to express gender variance. For birth-assigned adolescent males with GD, 3.0% were also diagnosed with ASD. The coincidence of GD with ADHD and ASD could be related to underlying symptoms of these neurodevelopmental disorders. In clinical practice, adolescents with ASD have always felt “strange” or “different” compared with their peers, and this “strangeness” may be attributed to feelings associated with GD (de Vries et al., 2010).

Schizophrenia was a comorbidity for both assigned males and females at birth in adolescents. Transgender feelings in patients with schizophrenia indicate their relatively high frequency (Baltieri & De Andrade, 2009). Studies in Europe and North America have suggested that approximately 40–45% of adolescents diagnosed with GD present with clinically significant psychopathology (Kaltiala-Heino et al., 2018). However, according to the Mental Health Act in Taiwan, minors cannot seek psychological help without parents’ consent. Therefore, this estimate for adolescents with GD and the comorbidities may be an underestimate.

The comorbidity seen in the highest percent of individuals ≥ 18 years of age with GD was for depression, which was with the same frequency seen in the adolescent group. We were able to show that the frequency in the group ≥ 18 years of age was significantly greater than the general population for both assigned males at birth and assigned females at birth. One explanation for this high incidence of depression may be due to the culture of Taiwan. People in Chinese cultures emphasize family, honor, and maintaining “face” (one’s reputation), and being LGBT or having other forms of GD remains a form of shame for one’s family (Wong, 2015) and this remains taboo for most people in Taiwan. The significantly higher prevalence of depression in Taiwanese patients diagnosed with GD ≥ 18 years of age suggests strategies should be implemented to provide social and mental health support that meets the needs of this population.

Our data about the prevalence of GD in Taiwan revealed the sex ratio in favor of designated males at birth was consistent over time, which differs from the change in sex ratio reported for most Western countries. One explanation for this finding might be due to the Chinese culture of Taiwan, which continues to adhere to traditional values. Traditional Chinese cultural values emphasize the male’s role in the family (Hsiao et al., 2006), and an individual’s personal identity is embedded in the social network and is dependent upon the appropriate behavior in his social role (Bedford & Hwang, 2003). Males who fail to maintain one’s identity in the social hierarchy may want to escape into female roles (Tsai, 2016).

This study had some limitations. First, the only variable for individuals with GD was sex assigned at birth and age, which is a limitation of how the database of the HWDC is organized. Second, gender affirming medical treatment is not reimbursed by the NHI; therefore, data for treatment (yes/no) data are not available in the HWDC. Third, Taiwan initiated the Gender Equity Education Act and promoted LGBT education earlier than other countries in Asia and was the first Asian country which legalized same-sex marriage. Our data may not reflect the situation of other Asian countries. Finally, we did not use a reference cohort to serve as a control when determining comorbidities. This was due to the difficulty in establishing what should be used as the matching reference when searching the data base by demographic factors such as age, gender, socioeconomic status, and characteristics of the parents. Future studies should include a reference cohort for comparing the prevalence of comorbidities in the general population.

**Fig. 5:**
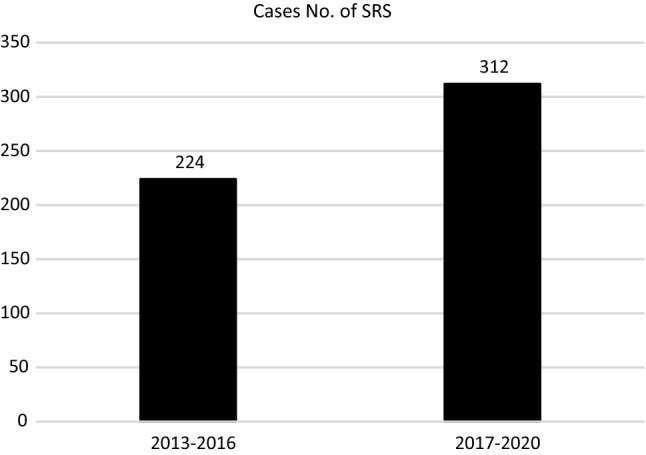
Numbers of individuals who underwent sex reassignment surgery and changed their gender identification in 2013–2016, and 2017–2020

Our findings demonstrate the number of assigned males at birth and assigned females at birth diagnosed with GD increased yearly from 2010 with the total number in 2019 twice that of 2010. These increases may be explained by the fact that more persons with GD are emboldened to seek help at clinics because of a friendlier environment regarding gender diversity. However, between 2013 and 2016, 224 persons underwent sex reassignment surgery (SRS) and changed their gender identification, with the number increasing to 312 persons (1.4 times higher) between 2017 and 2020 (Fig. 5) (Gender Equality Committee, 2021) in Taiwan.

A web survey conducted in 2020 in Taiwan reported 11.4% of the respondents identified as transgender (Du, 2020), which is a greater than the percentage in 2010 of 7.3% of assigned female young adults at birth and 1.9% of assigned male young adults at birth identifying as GD (Lai et al., 2010). One possible explanation for the increase that has occurred from 2010 to 2019 may be the combination of LGBT education and gender spectrum implemented in schools in Taiwan since 2012, as it has been suggested that education affirming transgender identification may help promote the idea that children may choose the opposite gender (Department for Education, 2020). However, these phenomena could also be due to adequate education about gender identity, which help youth recognize their feelings earlier and dare to come out earlier. The pros and cons of social and educational changes occurred in Taiwan need further validation.

Thus, further studies will be required to demonstrate whether there is a relationship between these variables and the increasing prevalence of GD demonstrated by our findings. We also recommend additional exploratory studies to obtain a better depiction of persons with GD in Taiwan. Conducting long-term quantitative and qualitative studies that include demographic and clinical variables, such as comorbidities, and personal experiences of living with GD are recommended to assess the impact on the psychological health and quality of life for persons with GD in Taiwan.

## Supplementary Information

Below is the link to the electronic supplementary material.

Supplementary file1 (DOCX 17 KB)

## Data Availability

The datasets generated and/or analyzed during the current study are available from the corresponding author on reasonable request.
