# Supplementary material for: Prevalence and Comorbidity of Gender Dysphoria in Taiwan, 2010–2019
Source: Arch Sex Behav. 2023 Jan 24;52(3):1009–17. doi: 10.1007/s10508-022-02500-7 (PMC10102133; doi:10.1007/s10508-022-02500-7)
Supplement: Supplementary file 1 — Supplementary file1 (DOCX 17 KB) [file 10508_2022_2500_MOESM1_ESM.docx]

**Supplemental Table 1**

*Diagnostic Codes for Attention-Deficit/Hyperactivity Disorder (ADHD), Autism Spectrum Disorder (ASD), Schizophrenia, and Depression*

|  | ICD-9-CM | ICD-10-CM |
| --- | --- | --- |
| *Schizophrenic disorders | 295,  295.0,295.00,295.01,295.02,295.03,295.04,295.05,  295.1,295.10,295.11,295.12,295.13,295.14,295.15,  295.2,295.20,295.21,295.22,295.23,295.24,295.25,  295.3,295.30,295.31,295.32,295.33,295.34,295.35,  295.4,295.40,295.41,295.42,295.43,295.44,295.45,  295.5,295.50,295.51,295.52,295.53,295.54,295.55,  295.6,295.60,295.61,295.62,295.63,295.64,295.65,  295.7,295.71,295.72,295.73,295.74,295.75,  295.8,295.8,295.81,295.82,295.83,295.84,295.85,  295.9,295.9,295.91,295.92,295.93,295.94,295.95 | F20,F20.0,F20.1,F20.2,F20.3,F20.5,F20.8,F20.81,F20.89,F20.9 |
| *Schizophrenic- related disorders | 297.0 ,297.1,297.2,297.3,297.8,297.9,  298,298,298.1,298.2,298.3,298.4,298.8,298.9 | F21,F22,F23,F24,  F25,F25.0,F25.1,F25.8,F25.9,F28,F29 |
|  |  |  |
| ASD | 299,299,299.8,299.9 | F84,F84.0,F84.2,F84.3,F84.5,F84.8,F84.9,F88,F89 |
|  |  |  |
| ADHD | 314,314.0,314,314.01,314.1,314.2,314.8,314.9 | F90,F90.0,F90.1,F90.2,F90.8,F90.9 |
|  |  |  |
| Depression | 296.21,296.22,296.23,296.24,  298.0,  296.25,296.26,296.82,296.20,311,  296.31,296.32,296.33,296.34,  298.0,296.30,  296.35,296.36,296.99,296.30,  301.10,301.13,300.4,301.12,296.99,296.99,296.90 | F32,  F32.0,F32.1,F32.2,F32.3,F32.4,F32.5,F32.8,F32.9,  F33,  F33.0,F33.1,F33.2,F33.3,  F33.4,F33.40,F33.41,F33.42,F33.8,F33.9,  F34,F34.0,F34.1,F34.8,F34.9,F39 |

^*^There is no separate code for “psychosis” in the National Health Insurance program for children. Therefore, the database of the Health and Welfare Data Science Center only contains ICD codes for “schizophrenia” no matter the age of the patient.
